# Supplementary material for: engGNN: A Dual-Graph Neural Network for Omics-Based Disease Classification and Feature Selection
Source: ArXiv. 2026 Jan 20:arXiv:2601.14536v1. Preprint. [Version 1] (PMC12869420)
Supplement: Supplement 1 [file NIHPP2601.14536v1-supplement-1.pdf]

A Supplemental Material

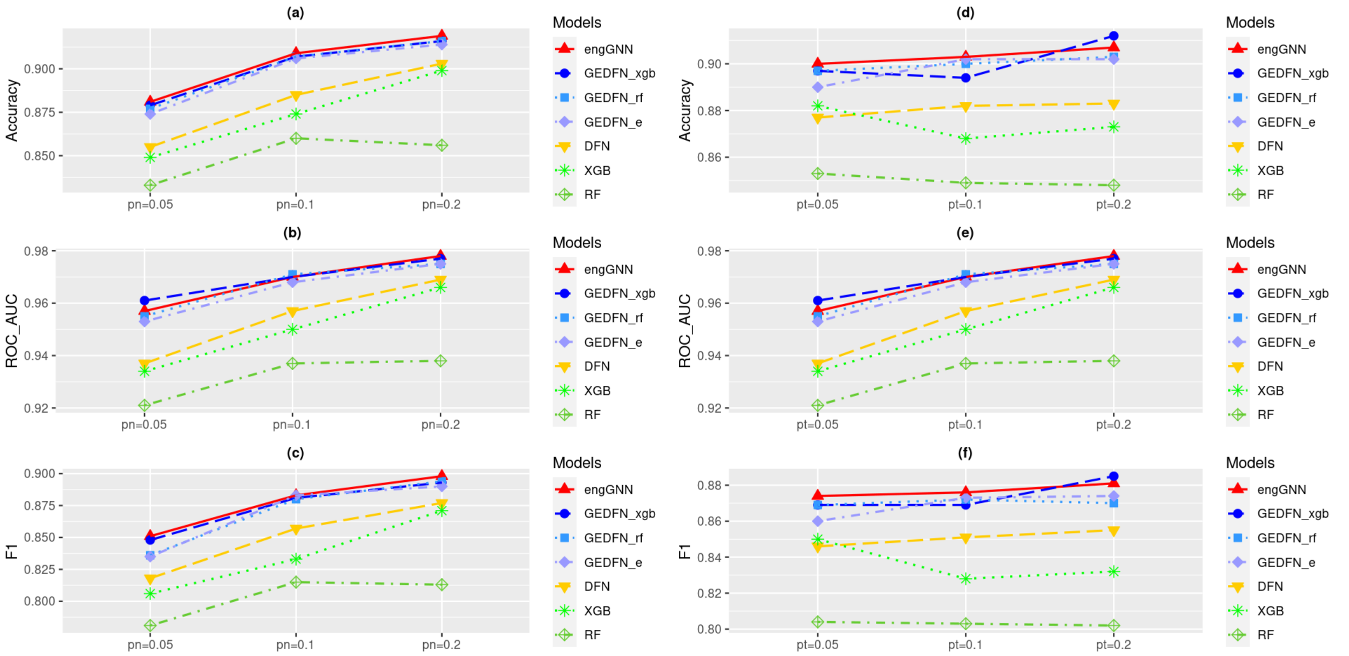

Supplemental Figure 1: Average classification performance of various models on simulated datasets across different values of sample size-to-feature size ratio (p_n) and proportion of true features (p_t). $p_n, p_t \in \{0.05, 0.1, 0.2\}$. Metrics include accuracy, ROC-AUC, and F1-score. Subplots (a) for different p_n values; Subplots (b) for different p_t values.

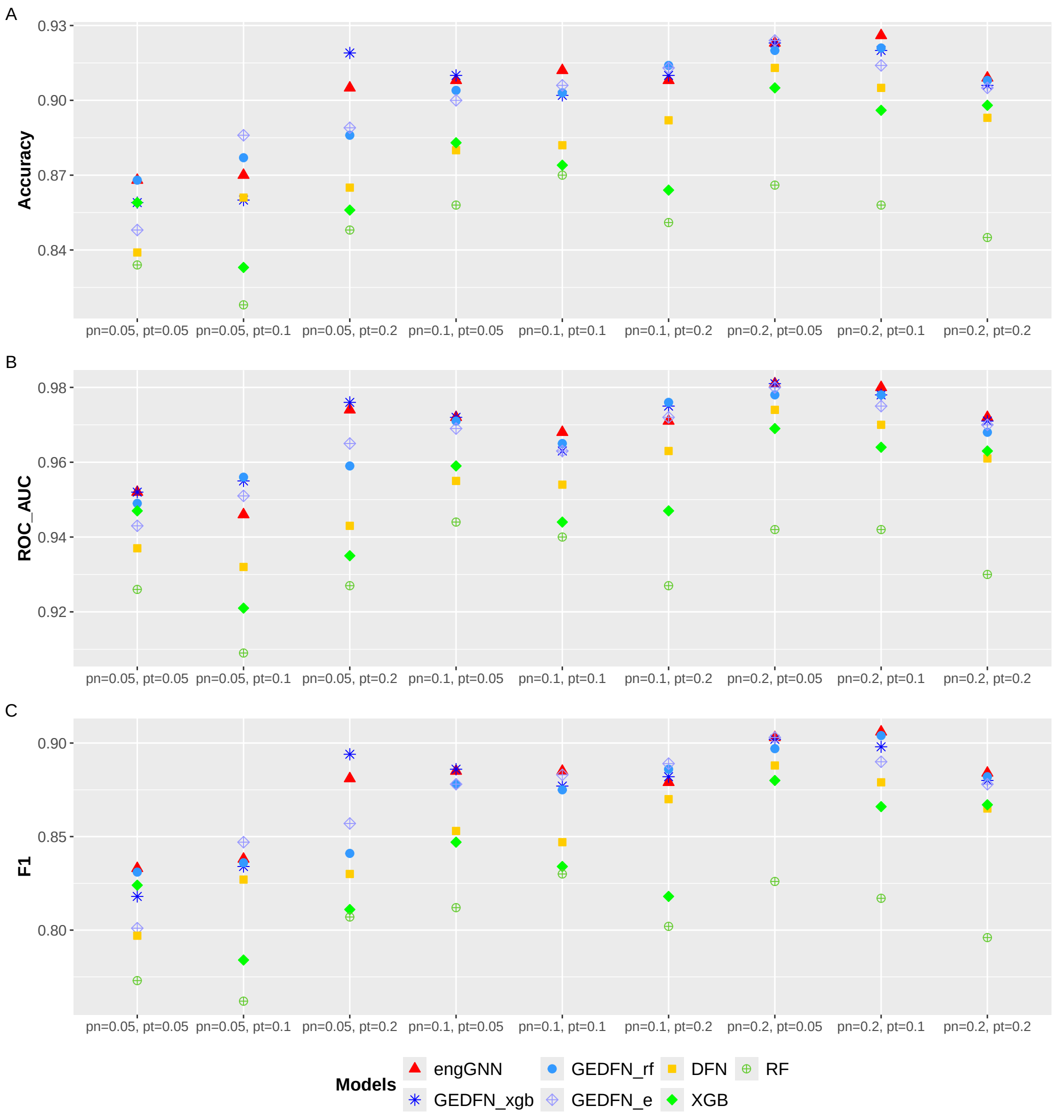

Supplemental Figure 2: Average classification performance of various models on simulated datasets across nine scenarios. The sample size is $n = 5000$, and each scenario was replicated 20 times.

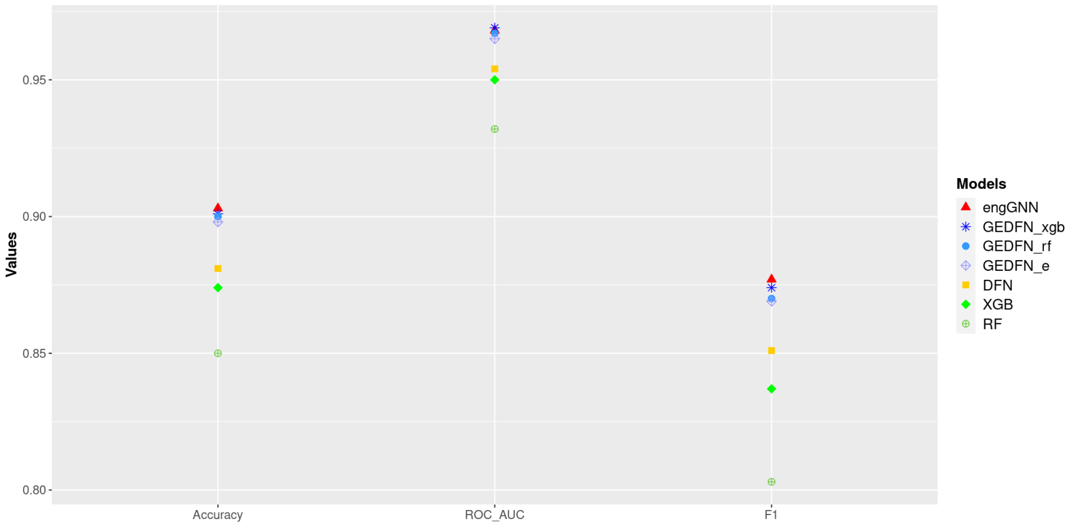

Supplemental Figure 3: Average classification accuracy, ROC-AUC, and F1-score of various models on simulated datasets across all the scenarios.

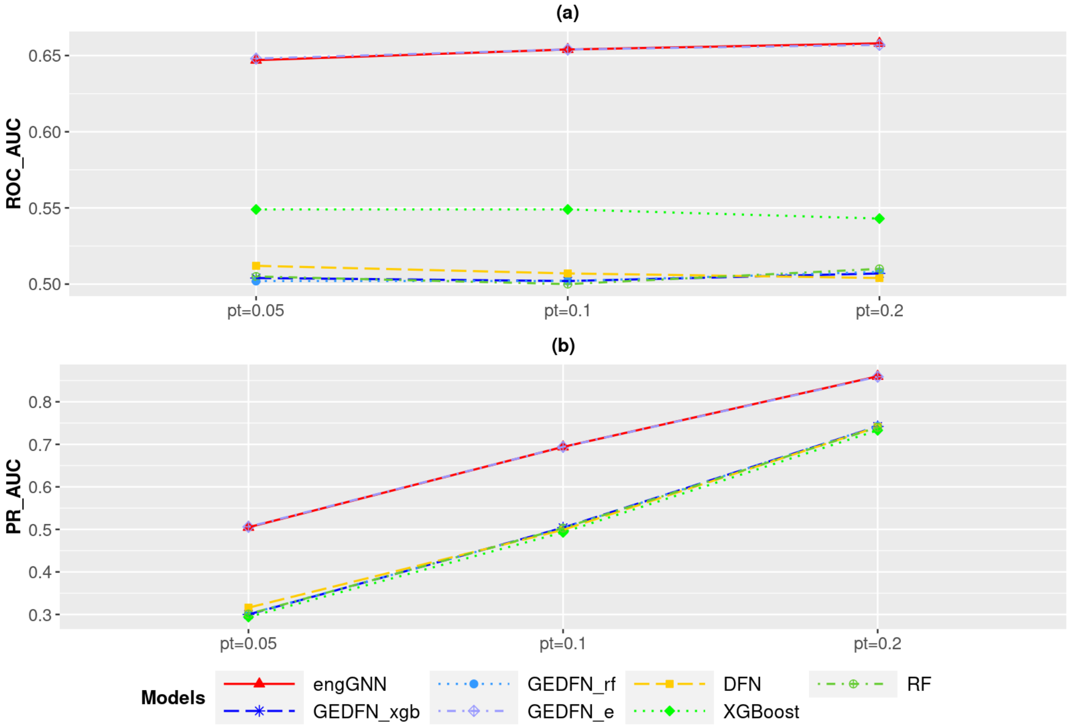

Supplemental Figure 4: Average feature selection performance of various models on simulated datasets across different proportions of true features ($p_t = 0.05, 0.1, 0.2$). Plot (a) ROC-AUC; Plot (b) PR-AUC.

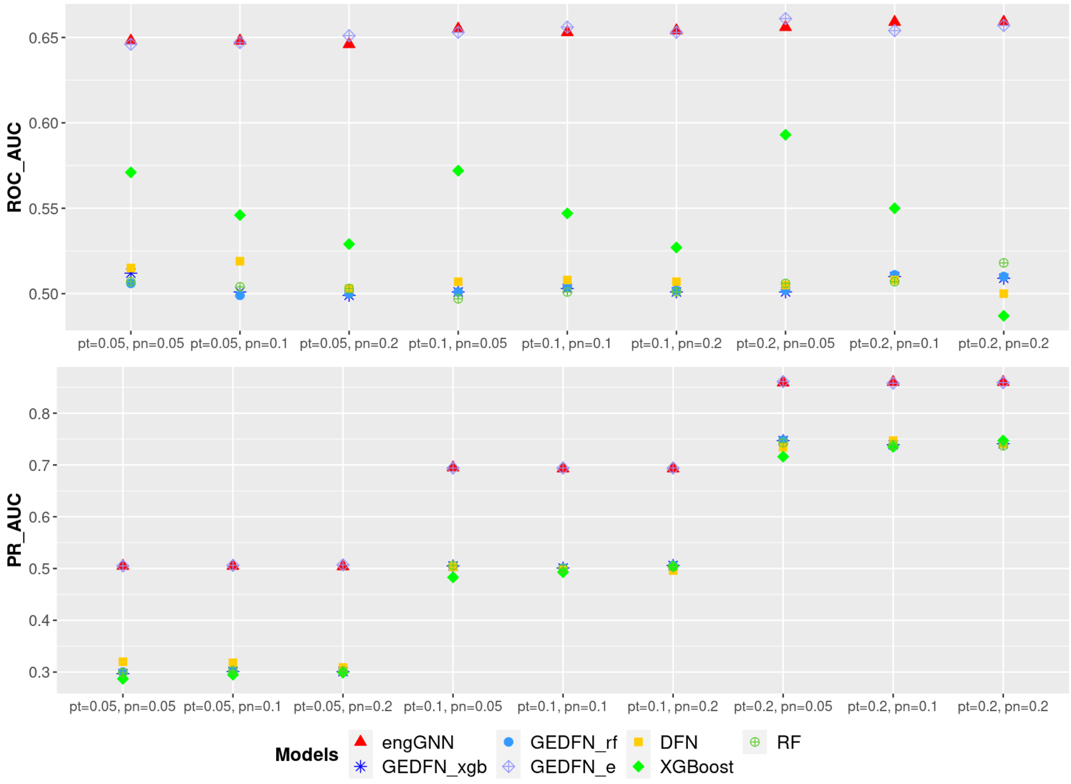

Supplemental Figure 5: Average feature selection performance of various models on simulated datasets across nine scenarios.

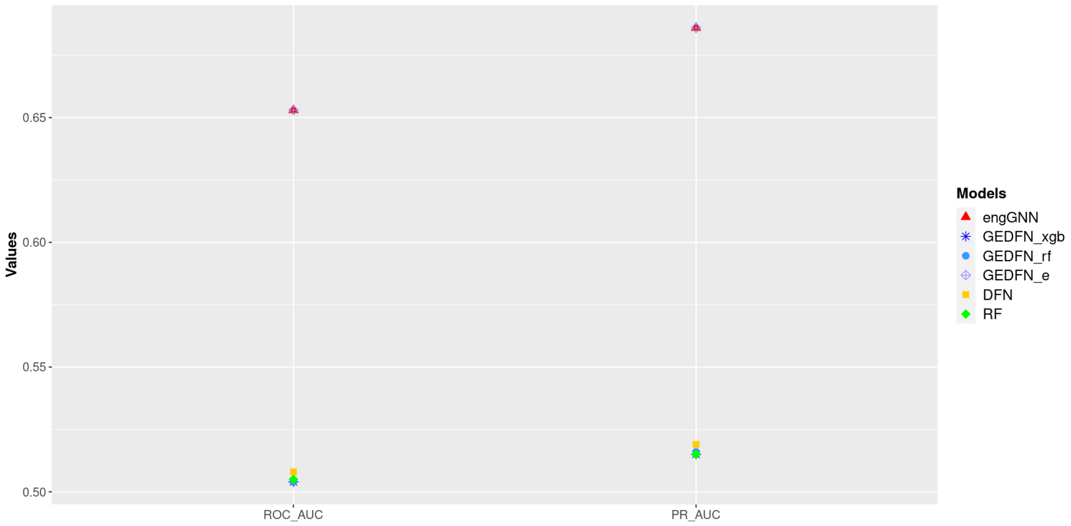

Supplemental Figure 6: Average feature selection ROC-AUC and PR-AUC of various models on simulated datasets across all the scenarios.

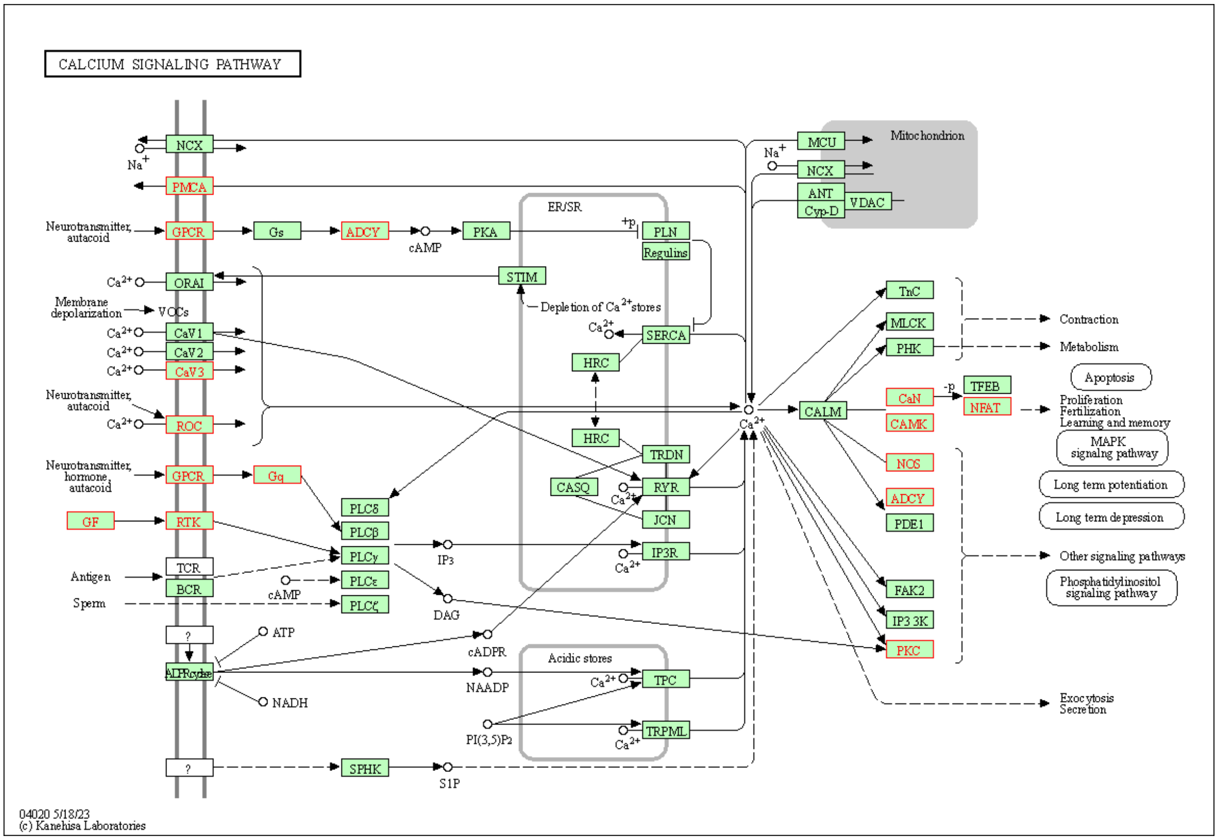

Supplemental Figure 7: Calcium signaling pathway

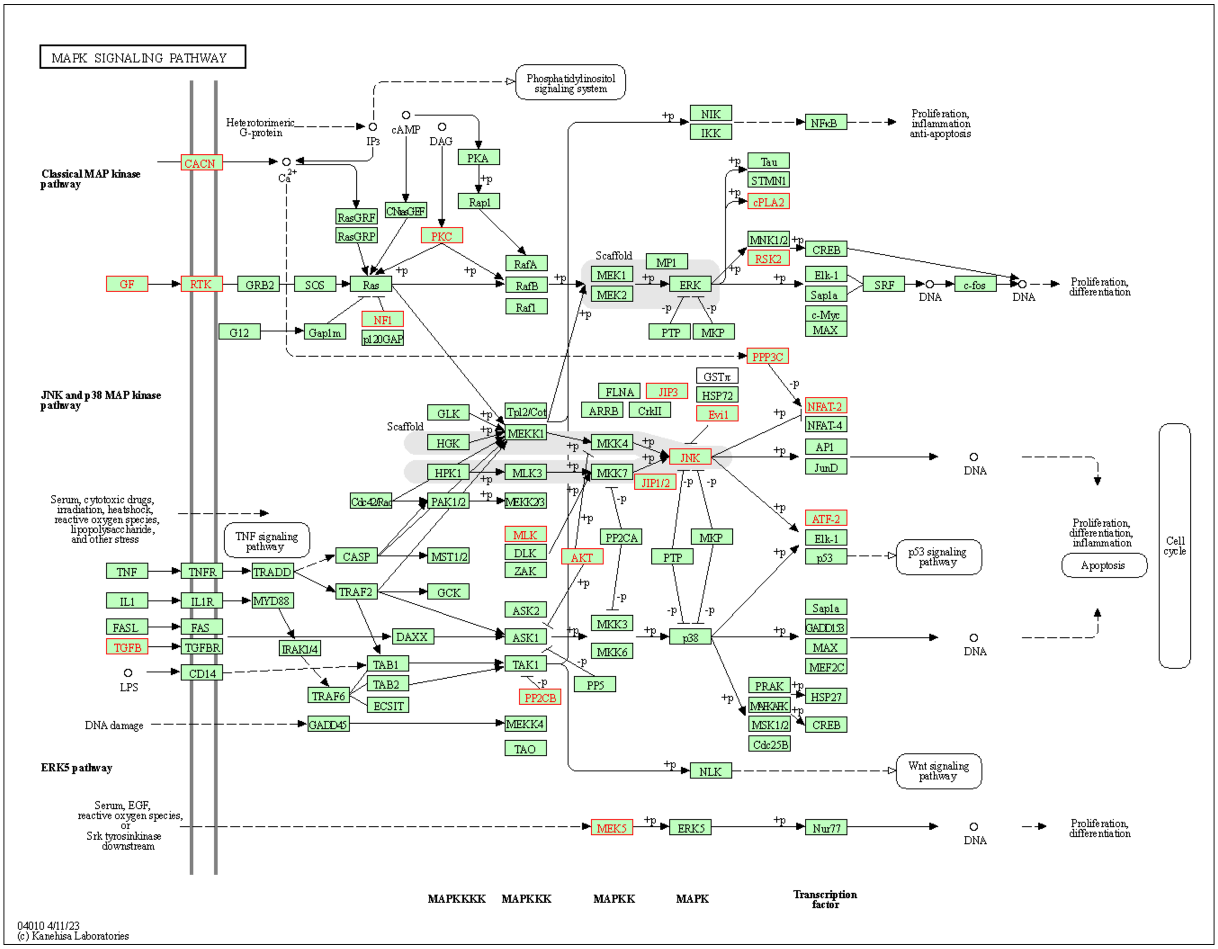

Supplemental Figure 8: MAPK signaling pathway

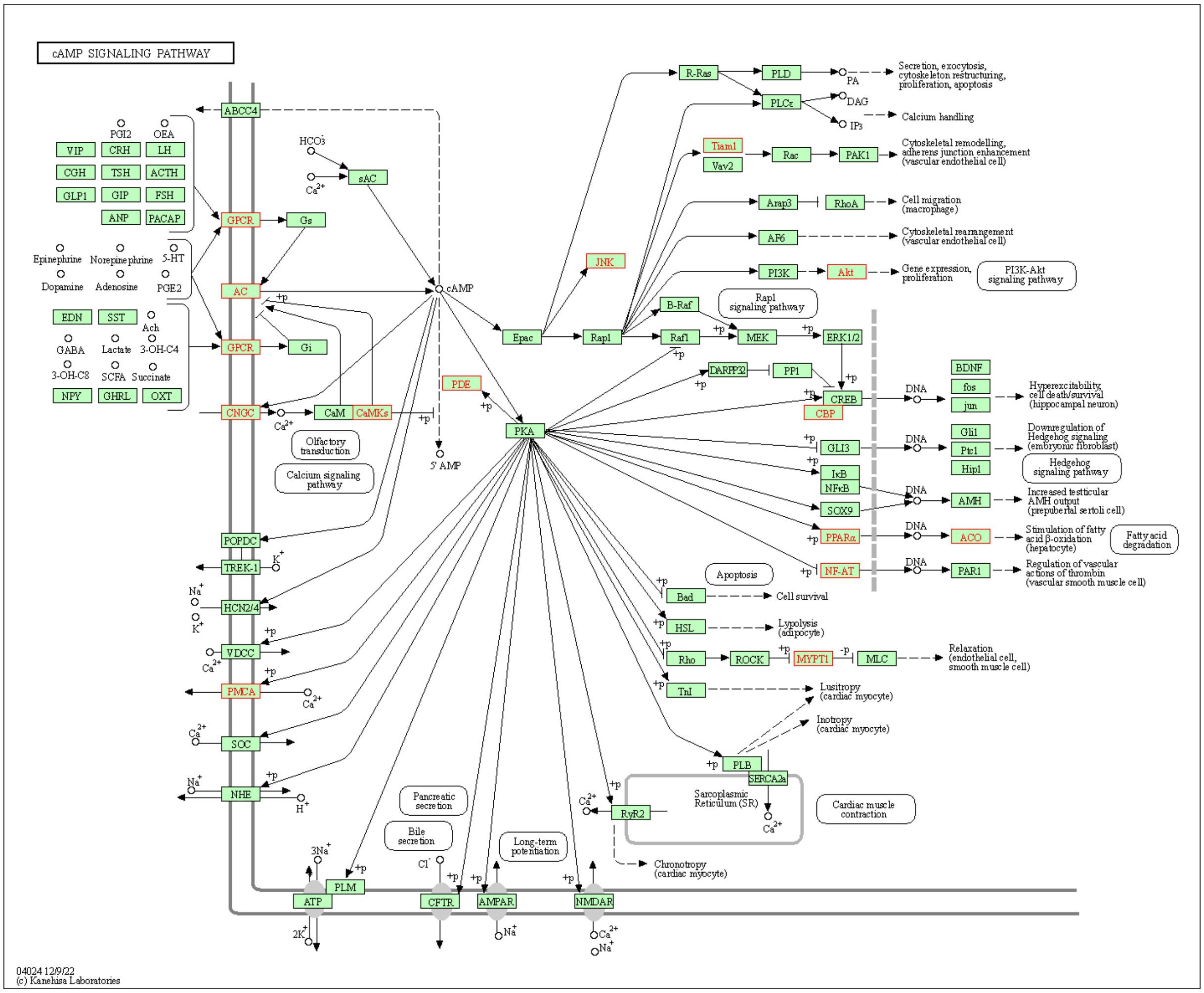

Supplemental Figure 9: cAMP signaling pathway

Supplemental Table 1: Hyperparameter tuning for engGNN on Simulated and Real Data. The optimal choices are bolded.

Hyperparameter	Tuning Range
Number of Trees in XGBoost	100, 1000, **0.2p**
Hidden neural size in GNN	(p, 64), (p, 128), (**p, 64, 16**), (p, 64, 32)
Hidden neural size in DFN	8, **16**, 32
Activation Function	**ReLU**
Learning Rate	**0.0001**
Batch Size	8, **16**, 32
Training Epochs	**50**, 100
Dropout Rate	**0.2**, 0.5

Supplemental Table 2: Feature selection performance (ROC-AUC and PR-AUC) across simulation scenarios with varying p_t (true feature proportion) and p_n (sample-to-feature ratio). The best-performing values in each row are bolded.

Metric	p_t	p_n	engGNN	$GEDFN_{xgb}$	$GEDFN_{rf}$	$GEDFN_{e}$	DFN	XGBoost	RF
ROC-AUC	0.05	0.05	**0.648**	0.512	0.506	0.646	0.515	0.571	0.507
	0.05	0.10	**0.648**	0.501	0.499	0.647	0.519	0.546	0.504
	0.05	0.20	0.646	0.499	0.500	**0.651**	0.503	0.529	0.503
	0.10	0.05	**0.655**	0.501	0.501	0.653	0.507	0.572	0.497
	0.10	0.10	0.653	0.503	0.504	**0.656**	0.508	0.547	0.501
	0.10	0.20	**0.654**	0.501	0.502	0.653	0.507	0.527	0.501
	0.20	0.05	0.656	0.501	0.502	**0.661**	0.505	0.593	0.506
	0.20	0.10	**0.659**	0.510	0.511	0.654	0.508	0.550	0.507
	0.20	0.20	**0.659**	0.509	0.510	0.657	0.500	0.487	0.518
PR-AUC	0.05	0.05	**0.505**	0.297	0.300	**0.505**	0.320	0.287	0.299
	0.05	0.10	0.505	0.301	0.301	**0.506**	0.318	0.295	0.303
	0.05	0.20	0.504	0.301	0.302	**0.507**	0.309	0.299	0.302
	0.10	0.05	**0.695**	0.505	0.503	0.694	0.503	0.483	0.506
	0.10	0.10	0.693	0.501	0.501	**0.694**	0.497	0.493	0.500
	0.10	0.20	0.693	0.506	0.506	**0.694**	0.496	0.504	0.503
	0.20	0.05	0.859	0.747	0.748	**0.861**	0.734	0.716	0.743
	0.20	0.10	**0.860**	0.739	0.739	0.858	0.747	0.735	0.739
	0.20	0.20	**0.860**	0.741	0.741	0.859	0.741	0.747	0.737
